# In vivo measurement of apolipoprotein E from the brain interstitial fluid using microdialysis

**DOI:** 10.1186/1750-1326-8-13

**Published:** 2013-04-19

**Authors:** Jason D Ulrich, Jack M Burchett, Jessica L Restivo, Dorothy R Schuler, Philip B Verghese, Thomas E Mahan, Gary E Landreth, Joseph M Castellano, Hong Jiang, John R Cirrito, David M Holtzman

**Affiliations:** 1 Department of Neurology, Saint Louis, MO, USA; 2 Hope Center for Neurological Disorders, Saint Louis, MO, USA; 3 Developmental Biology, Saint Louis, MO, USA; 4 Knight Alzheimer’s Disease Research Center, Washington University School of Medicine, Saint Louis, MO, 63110, USA; 5 Department of Neurosciences, Case Western Reserve University, School of Medicine, Cleveland, OH, 44106USA

**Keywords:** Microdialysis, Apolipoprotein E, Alzheimer’s disease

## Abstract

**Background:**

The *APOE4* allele variant is the strongest known genetic risk factor for developing late-onset Alzheimer’s disease. The link between apolipoprotein E (apoE) and Alzheimer’s disease is likely due in large part to the impact of apoE on the metabolism of amyloid β (Aβ) within the brain. Manipulation of apoE levels and lipidation within the brain has been proposed as a therapeutic target for the treatment of Alzheimer’s disease. However, we know little about the dynamic regulation of apoE levels and lipidation within the central nervous system. We have developed an assay to measure apoE levels in the brain interstitial fluid of awake and freely moving mice using large molecular weight cut-off microdialysis probes.

**Results:**

We were able to recover apoE using microdialysis from human cerebrospinal fluid (CSF) *in vitro* and mouse brain parenchyma *in vivo*. Microdialysis probes were inserted into the hippocampus of wild-type mice and interstitial fluid was collected for 36 hours. Levels of apoE within the microdialysis samples were determined by ELISA. The levels of apoE were found to be relatively stable over 36 hours. No apoE was detected in microdialysis samples from apoE KO mice. Administration of the RXR agonist bexarotene increased ISF apoE levels while ISF Aβ levels were decreased. Extrapolation to zero-flow analysis allowed us to determine the absolute recoverable concentration of apoE3 in the brain ISF of apoE3 KI mice. Furthermore, analysis of microdialysis samples by non-denaturing gel electrophoresis determined lipidated apoE particles in microdialysis samples were consistent in size with apoE particles from CSF. Finally, we found that the concentration of apoE in the brain ISF was dependent upon apoE isoform in human apoE KI mice, following the pattern apoE2>apoE3>apoE4.

**Conclusions:**

We are able to collect lipidated apoE from the brain of awake and freely moving mice and monitor apoE levels over the course of several hours from a single mouse. Our technique enables assessment of brain apoE dynamics under physiological and pathophysiological conditions and in response to therapeutic interventions designed to affect apoE levels and lipidation within the brain.

## Background

Intercellular lipid transport in the central nervous system (CNS) is predominantly mediated by high-density lipoprotein (HDL)-like particles containing the lipid-binding protein apolipoprotein E (apoE) [1]. Within the CNS, apoE is secreted primarily by astrocytes [2]. In humans there are three common *APOE* allele variants: *APOE2*, *APOE3*, and *APOE4*. Possession of the *ϵ4* allele is the strongest known genetic risk factor for developing late-onset Alzheimer’s disease (LOAD), while apoE2 is protective[2]. ApoE likely influences AD in large part through the isoform-dependent effects of apoE on the metabolism of amyloid-β (Aβ) in the brain. ApoE isoforms differentially influence the rate of Aβ clearance from the brain with the order of clearance being apoE4<apoE3<apoE2 [3]. ApoE also facilitates the formation of Aβ aggregates (e.g. oligomers and fibrils) within the brain, which are thought to induce synaptic and neuronal toxicity [4]. In addition to isoform-dependent effects, the degree of apoE lipidation and apoE concentration within the brain also influences Aβ plaque deposition and Aβ clearance [5-8]. ApoE likely plays additional roles in the brain in processes such as synaptic plasticity, regulation of extracellular lipid metabolism, and inflammation (for review, see [2]).

Despite the importance of apoE in AD, little is known about the physiological regulation of apoE within the CNS. To date, analysis of the regulation of apoE expression and lipidation within the CNS has been limited to *in vitro* cell culture systems and assessment of apoE from homogenized brain extracts or cerebral spinal fluid (CSF). Thus, very little is known about the dynamic regulation of apoE within the brain, particularly within the interstitial fluid (ISF), where apoE is present immediately after secretion from astrocytes and where it likely functionally interacts with Aβ and other molecules. Given recent interest in apoE as a therapeutic target for AD, there is a critical need to develop methods to dynamically assess the levels, lipidation, and effects of apoE within the brain *in vivo*[8,9].

Microdialysis is a widely used and powerful technique that has been employed to study neurotransmitters and other small molecules or peptides within the brain [10]. In AD, microdialysis has been instrumental in studying the dynamics of Aβ production and clearance *in vivo*[3,11-14]. However, the comparatively large size of lipidated apoE particles (~300 kDa) has impeded the utility of microdialysis to similarly assess apoE metabolism within the brain. A recent report demonstrated that a high molecular weight cut-off (MWCO) microdialysis probe was capable of collecting small (<30 kDa) proteins from the brain ISF[15]. We hypothesized that such a probe might be useful for collecting lipidated lipoproteins. Here, we report the use of the high MWCO AtmosLM microdialysis probe to collect and quantify lipidated apoE particles from the brain ISF of awake and freely moving animals by *in vivo* microdialysis. Non-denaturing gel electrophoresis analysis of the ISF samples indicates that apoE-containing particles are heterogeneous in size and are consistent in size with apoE-containing particles from CSF. Using this technique, we found that the level of apoE in the hippocampal ISF is dependent upon apoE isoform. The ability to dynamically measure apoE levels and lipidation in the brain ISF will be useful for studying lipid metabolism within the brain ISF and the molecular basis for the effect of apoE on AD pathology.

## Results and discussion

**Figure 1:**
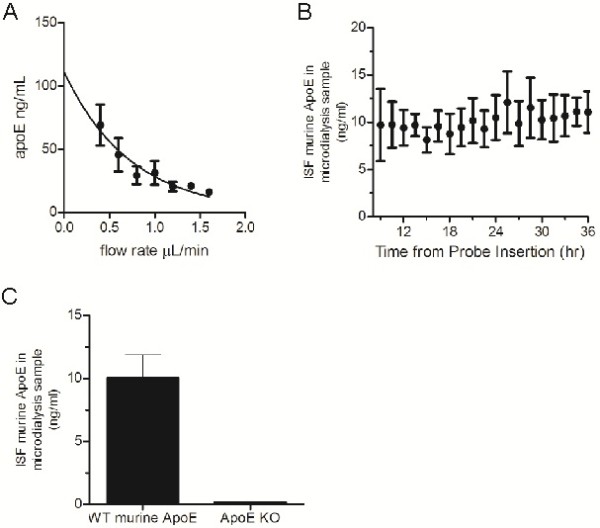
**Analysis of apoE levels by microdialysis *in vitro *and *in vivo*. A.** Microdialysis samples were collected hourly from human CSF *in vitro* at flow rates ranging from 0.4 μL/min to 1.6 μL/min. The concentration of apoE within microdialysis samples and CSF was determined by ELISA. Data points represent mean ± SEM (n=3). A single-phase exponential decay curve (r^2^=0.93) was used to calculate the estimated mean concentration of apoE at zero flow. **B.** Microdialysis probes were implanted into the hippocampus of 3-4 month old mice and ISF collected bi-hourly for 36 hours. The concentration of apoE within microdialysis samples was determined by ELISA. Data points represent mean ± SEM (n=5). **C.** The mean concentration of murine apoE in ISF microdialysis samples from WT (n=5) and apoE KO mice (n=2) was determined by ELISA. ISF samples were collected at a constant flow rate of 1.0 μL/min. Data are presented as mean ± SEM.

The relatively large hydrated diameter of lipidated apoE particles prohibits collection by commonly used lower MWCO microdialysis probes (typically 6-40 kDa). To circumvent this issue, we optimized the use of a 1,000 kDa MWCO microdialysis probe for collection of apoE. We first characterized the efficiency of apoE collection from human CSF using microdialysis *in vitro*. The recovery of analyte by microdialysis is inversely related to the flow rate through the microdialysis probe[10]. By extrapolating along the recovery curve to a zero flow rate, it is possible to estimate the maximum amount of exchangeable analyte [10]. Microdialysis samples were collected hourly at flow rates ranging from 0.4 μL/min to 1.6 μL/min. As expected, decreasing the flow rate increased the amount of apoE detected in microdialysis samples (Figure 1A). We estimated the total amount of exchangeable apoE to be 111.2 ± 14.4 ng/mL, which represented ~2% of the 6024 ± 152.3 ng/mL total apoE detected within the CSF samples.

Having determined it was possible to collect apoE by microdialysis using a 1,000 kDa MWCO probe, we next tested whether we could collect apoE *in vivo*. We implanted microdialysis probes into the hippocampus of 3-4 month old wild-type mice and collected microdialysis samples every two hours at a constant flow rate of 1.0 μL/min. Under these conditions, levels of apoE within the hippocampal ISF were stable throughout the 36 hour collection duration (Figure 1B). Similar to previous studies, we observed only minimal astrogliosis in the cortex proximal to the site of cannula insertion and no evidence of substantial inflammation following microdialysis (data not shown)[14,16]. We found the mean concentration of recoverable apoE in the hippocampal ISF to be 10.1 ± 1.8 ng/mL at a constant flow rate of 1.0 μL/min (Figure 1C). To verify the specificity of our assay we also performed microdialysis in apoE KO mice. As expected, we detected no apoE in the apoE KO mouse ISF dialysate (Figure 1C). These data confirm that we are able to collect apoE from the brain ISF and specifically measure how it changes over the course of several hours in a single mouse.

Due to the relatively high variance we observed in apoE levels, we performed a power analysis to determine if we could feasibly detect statistically significant differences in ISF apoE levels. Given the observed mean concentration and average standard deviation of murine ISF apoE at a given time point, we estimate ~5-6 animals per group would be required to observe a statistically significant 70% change in apoE levels at an α-value of 0.80 and p-value of 0.05. Furthermore, because *in vivo* microdialysis enables assessment of changes in apoE levels in a single mouse, normalizing the level of apoE detected to a baseline value (i.e. the mean level from first 9 hours of collection) for each animal eliminates inter-animal variability in apoE levels and allows for detection of a statistically significant 50% change in relative apoE levels with ~5-6 animals.

**Figure 2:**
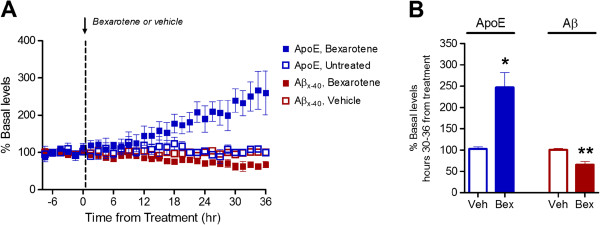
**Bexarotene increases ISF apoE levels and decreases ISF Aβ levels. A.** ISF Aβ_x-40_ and apoE levels in the hippocampus of 2-month old APP/PS1 mice were monitored using *in vivo* microdialysis. Following establishment of a 6 hour baseline ISF level for Aβ_x-40_ and apoE, mice were administered bexarotene (100 mg/kg p.o.) or vehicle (water) and ISF Aβ_x-40_ and apoE levels assessed for an additional 36 hours. **B.** The mean percent change from baseline of ISF apoE and Aβ_x-40_ levels 30-36 post-administration was compared between vehicle and bexarotene treated mice. Bexarotene significantly increased ISF apoE levels (247 ± 34.3%, n=2) compared to vehicle (103 ± 5.2%, n=3) (*p<0.05, unpaired t-test). Bexarotene decreased ISF Aβ levels (65.1 ± 8.1%, n=3) compared to vehicle (100 ± 3.7%, n=6). (**p<0.005, unpaired t-test).

Recent studies propose that modulating apoE levels and lipidation in the brain using nuclear hormone receptor agonists may be an effective therapeutic target for AD [8,17]. We tested whether we could detect changes in ISF apoE and Aβ levels following administration of the retinoid-X-receptor (RXR) agonist bexarotene using microdialysis. We monitored hippocampal ISF apoE and Aβ levels in 2-month old APP/PS1 mice. Following establishment of a 6-hour baseline apoE and Aβ level, bexarotene (100 mg/kg) or vehicle (water) were administered to the mice via oral gavage. Bexarotene treatment led to a steady increase in ISF apoE levels beginning ~12 hours post-administration (Figure 2A). ISF apoE levels were increased 2.5-fold 30-36 hours post-treatment (Figure 2A and B). ISF Aβ levels were decreased by ~35%, similar to previous observations (Figure 2A and B) [8]. These data demonstrate the utility of microdialysis to detect biologically relevant, pharmacologically-induced changes in ISF apoE levels.

**Figure 3:**
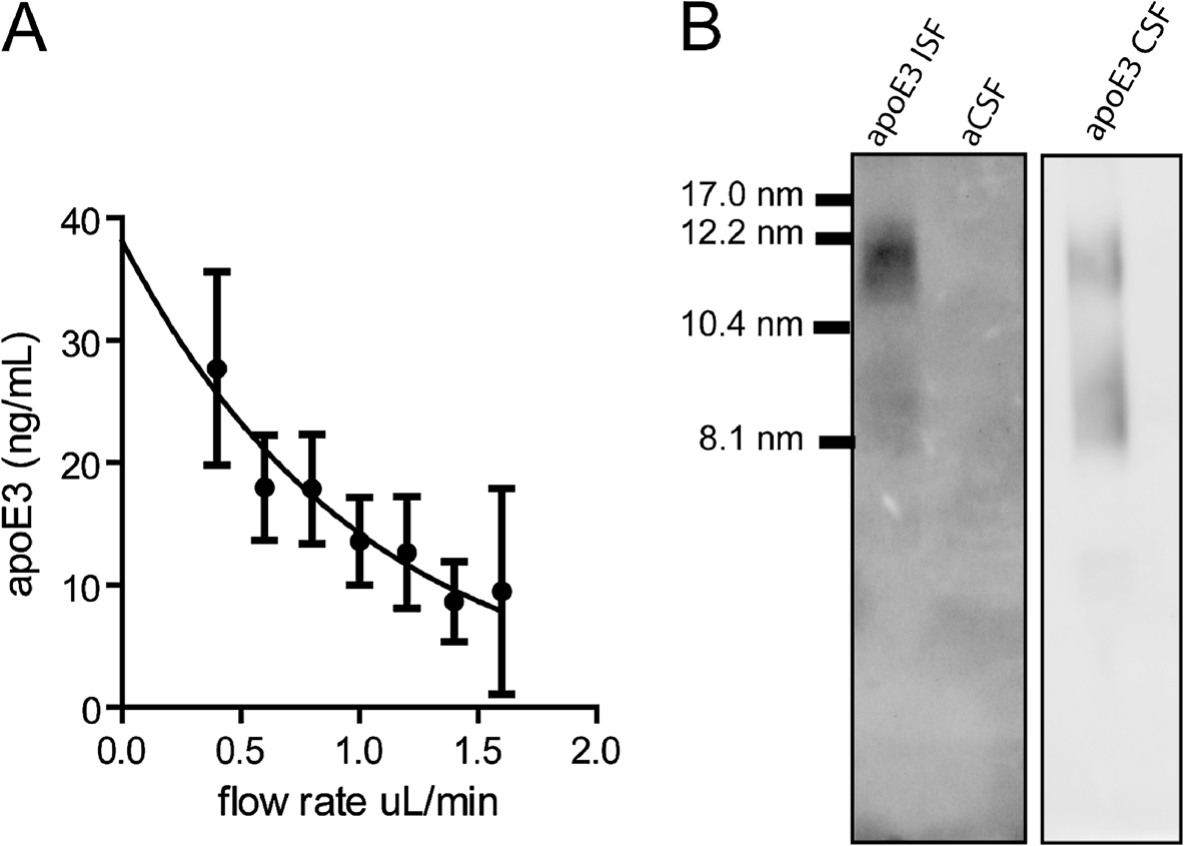
**Analysis of hippocampal ISF apoE3 levels and lipidation. A.** The apoE concentration in microdialysis samples collected at flow rates ranging from 0.4 μL/min to 1.6 μL/min was determined by ELISA. Data are presented as mean ± SEM (n=4). A single-phase exponential decay curve (r^2^=0.93) was used to calculate the estimated mean concentration of apoE3 at zero flow. **B.** The lipidation of apoE in ISF and CSF samples was analyzed by non-denaturing gel electrophoresis using 4-20% Tris-glycine gradient gels. No apoE was detected in the aCSF used for microdialysis. The samples were run on the same gel; however the CSF panel is from a shorter exposure than the ISF panel for clarity.

To further validate our technique we tested whether we could determine an absolute concentration of recoverable apoE within the hippocampal ISF by the extrapolated zero-flow method. We implanted microdialysis probes into the hippocampus of 3-4 month old apoE3 KI mice and collected ISF dialysate at flow rates ranging from 0.4 μL/min to 1.6 μL/min. We then fit a single-exponential decay curve to the concentration of apoE as a function of flow rate (Figure 3A). Our analysis determined the concentration of recoverable apoE3 in the ISF under steady-state conditions was 38.1 ± 4.0 ng/mL (n=4).

ApoE particles in the CSF or secreted by cultured astrocytes are a heterogeneous mixture that vary in size depending upon the degree of lipidation [5,18-20]. We compared the lipidation of apoE3 particles collected from brain parenchymal ISF to apoE3 particles in CSF by non-denaturing gel electrophoresis, which separates proteins based upon their hydrated diameter. As previously reported, apoE3 particles in CSF were heterogeneous in size, ranging from 8.1 nm to 17.0 nm (Figure 2B) [21]. ApoE3 particles collected from ISF were lipidated and were similar in size to particles found in CSF (Figure 3B). We further verified that the lipidated apoE3 particles in ISF samples were not due to contamination from the artificial cerebral spinal fluid (aCSF) used for microdialysis (Figure 3B).

**Figure 4:**
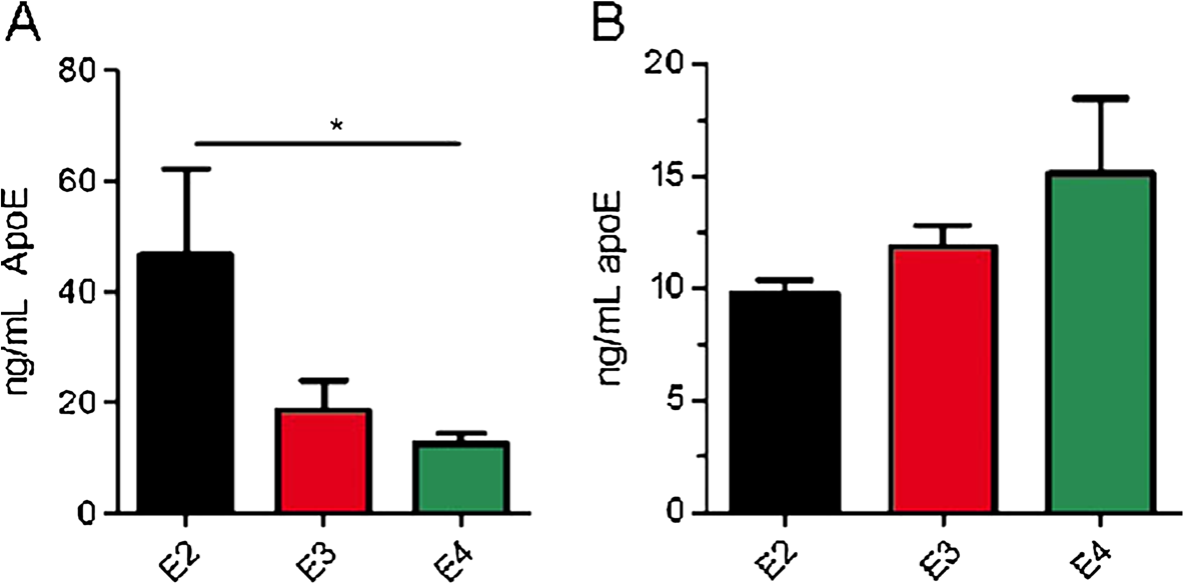
**Isoform-dependent differences in apoE levels in the hippocampal ISF. A.** ISF samples from the hippocampus of apoE2 KI, apoE3 KI, and apoE4 KI mice were obtained by microdialysis using a constant flow-rate of 1.0 μL/min. ApoE levels were assessed by ELISA and compared by ANOVA followed by Tukey’s post hoc test. * p<0.05, n=3-6 mice per genotype. Data are presented as mean ± SEM. **B.** 12.5 ng/mL of recombinant apoE2, apoE3, or apoE4 was measured by ELISA and compared by ANOVA (p=0.22).

Previous studies found an isoform-dependent effect on apoE levels in the hippocampus, cortex, and CSF in apoE KI mice with apoE2-expressing mice having the highest levels of apoE and apoE4 expressing mice the lowest [22,23]. Therefore, we tested whether we could detect isoform-dependent differences in apoE levels in the hippocampal ISF by microdialysis. We implanted microdialysis probes into the hippocampus of 3-4 month old apoE2, apoE3 or apoE4 KI mice and assessed the absolute concentration of human apoE in the ISF dialysate by ELISA. The concentration of apoE2 within the ISF (46.7 ± 15.6 ng/mL, n=3) was significantly greater than that of apoE4 (12.6 ± 1.8 ng/mL, n=4) and the concentration of apoE3 (18.7 ± 5.3, n=6) was at a level between that of apoE2 and apoE4 (Figure 4A). To confirm that the observed differences were not due to differential detection of apoE isoforms by the antibodies used in the ELISA, we measured equal amounts of recombinant apoE2, apoE3, and apoE4 by ELISA and found no significant difference in detection among the apoE isoforms (Figure 4B). Therefore, these results support the utility of our assay for observing differences in apoE levels in the ISF that are similar to those seen in CSF and in the brain.

## Conclusions

We have developed a sensitive microdialysis assay for assessing the levels and lipidation of apoE within the brain parenchymal ISF in awake and freely moving animals. Despite being the strongest genetic risk factor for developing late-onset AD, the precise molecular mechanism by which *APOE* genotype influences the risk of developing AD remains unknown. Previous studies indicate that apoE affects the metabolism and oligomerization of Aβ within the brain [3,4,24] as well as influences a variety of other biological processes [2]. Recent studies have therapeutically targeted the levels and lipidation of apoE to enhance clearance of Aβ from the brain [8]. The method described here will be useful in sampling ISF apoE levels over time in order to assess the relationship between apoE levels and lipidation and Aβ metabolism in the brain. Furthermore, assessment of apoE by microdialysis could prove useful in investigating the regulation of extracellular lipid homeostasis within the brain. This may be important in synaptic plasticity and repair after brain injury, as well as potentially play a role in neurodegenerative diseases such as Niemann-Pick disease [25].

## Methods

### Materials

Mouse apoE monoclonal antibodies (mHJ 6.1, mHJ 6.2, and mHJ 6.3) were generated in-house and have been described previously [9]. Polyclonal apoE antibody (50A-G1b) was purchased from Academy Bio-medical Co. (Houston, TX, USA). Recombinant apoE3 was purchased from Leinco Technologies (St. Louis, MO, USA). Bexarotene (Targretin™) was provided by Dr. Gary Landreth (Case Western Reserve University).

### Animals

ApoE2, apoE3, and apoE4 knock-in (KI) mice were generously provided by Dr. Patrick M. Sullivan (Duke University) and have been previously described [26]. To assess murine apoE, wild type mice on a mixed C57BL6/C3H background were utilized (Jackson Labs). APPswe/PS1δE9 mice (APP/PS1) and ApoE knock-out (KO) mice were obtained from Jackson Labs. Mice were housed under constant light/dark conditions and had access to food and water *ad libitum*. All experimental protocols were approved by the Animal Studies Committee at Washington University in St. Louis.

### *In vitro* microdialysis

Human CSF was collected by lumbar puncture from cognitively normal research volunteers from the Washington University Memory and Aging Project [27]. 1,000 kDa MWCO membrane microdialysis probes (AtmosLM Microdialysis probe, PEP-X-0Y, Eicom, San Diego, CA, USA) were connected to a two-channel peristaltic push-pull pump (MAB20, SciPro, Sanborn, NY, USA). The inlet-port of the microdialysis probe was connected to the push channel of the pump using Joint Teflon Tubing (inner diameter 0.1 mm) (JT-10-100, Eicom, San Diego, CA, USA) and the outlet-port was connected to the pull channel of the pump using Teflon (FEP) tubing (inner diameter 0.12 mm). The flow rates of the push and pull channels were calibrated to within 5% tolerance. The probes were then flushed with microdialysis perfusion solution (4% human albumin (Gemini Bioproducts, West Sacramento, CA, USA)-containing artificial CSF (aCSF) (in mM: 1.3 CaCl_2_, 1.2 MgSO_4_, 3 KCl, 0.4 KH_2_PO_4_, 25 NaHCO_3_, 122 NaCl, pH 7.35) that had been prepared on the day of use and filtered through a 0.1 μm PES membrane. Microdialysis was performed at flow rates of 0.4 μL/min to 1.6 μL/min and samples were collected hourly using a refrigerated fraction collector (Univentor 820 Microsampler, SciPro). Extrapolation from zero-flow analysis was performed by fitting single-phase exponential decay curve to the mean apoE concentrations at varying flow rates with the constraint that the plateau was set to 0. Percent recovery for each flow rate was calculated using *C*_*x*_/E * 100, where *C*_*x*_ was the apoE concentration at a given flow rate and *E* was the estimated apoE concentration at steady-state.

### *In vivo* microdialysis

Mice were anesthetized using 1.5%-2.5% isoflurane, the head shaved, and an anterior to posterior incision made along the midline of the head to expose the skull from several mm anterior of bregma to several mm posterior of lambda. The mouse was then mounted onto a manipulator arm-equipped small animal stereotaxic apparatus (David Kopf Instruments, Tujunga, CA, USA). The skull was then leveled to within 0.1 mm at lambda, bregma, and two points 2.2 mm lateral of midline. A bore hole (1.0 mm diameter) was then created above the left hippocampus (bregma -3.1 mm, 2.5 mm lateral, dura mater -0.6 mm). A second bore hole (0.75 mm) was placed in the right, anterior quadrant of the skull in which to place an anchoring bone screw. An AtmosLM Guide Cannula (PEG-X, Eicom, San Diego, CA, USA) was then stereotactically inserted into the left hippocampal formation (12° angle, dura mater -1.2 mm). The cannula was then secured into place using a binary dental cement. An AtmosLM Dummy Cannula (PED-X, Eicom, San Diego, CA, USA) was then inserted into the guide cannula and secured with a plastic cap nut. The wound was then closed using surgical adhesive and the animal placed into a clean cage and provided with access to food and water *ad libitum*.

1,000 kDa microdialysis probes (AtmosLM Microdialysis probe, PEP-X-0Y, Eicom, San Diego, CA, USA) were prepared as described above. Mice were then briefly anesthetized with isoflurane and probes were inserted into the hippocampus through the guide cannula and the mouse fitted with a plastic collar. Mice were then placed into a cage designed to allow for free movement without placing stress on the Teflon tubing or probe apparatus (Raturn Stand-Alone System, BASi, West Lafayette, IN, USA). The mice were kept under constant light conditions for the remainder of the experiment. Peristaltic pumps were operated at maximum speed for two hours to prevent clogging of the microdialysis membrane. Flow rates were then reduced to 1.0 μL/min to measure apoE. Samples were collected bi-hourly using a refrigerated fraction collector (Univentor 820 Microsampler, SciPro).

### ApoE and Aβ ELISAs

The apoE concentration in microdialysis samples were analyzed by an apoE sandwich ELISA. For human apoE, a 96-well plate (Nunc) was coated with 500 ng/well HJ 6.2 overnight at 4°C. For murine apoE, 1% milk served as the capture ligand. The plate was then blocked with 1% milk for 60 min. at 37°C. Microdialysis samples were diluted with our standard ELISA buffer (0.5% BSA, 0.025% Tween-20 in PBS, pH 7.4). For human apoE isoforms recombinant apoE3 standards were diluted with ELISA buffer and microdialysis perfusion buffer to match the buffer composition of microdialysis samples. For quantification of murine apoE, pooled C57BL/6J plasma was used as a standard. Samples and standards were loaded and incubated overnight at 4°C. Biotinylated mHJ 6.3 or mHJ 6.1 was used as a detection antibody for mouse or human apoE, respectively. Following a 90 min. incubation at 37°C with detection antibody, poly-streptavidin-horseradish peroxidase (HRP) (Thermo Scientific) was applied to the plate and incubated for 90 min. at room temperature. ELISAs were then developed using Super Slow ELISA TMB (Sigma) and read using a Bio-Tek FL-600 plate reader at 650 nm. Aβ_x-40_ was analyzed by sandwich ELISA as described previously, using HJ 2 as the capture antibody and biotinylated HJ 5.1 as the detection antibody [8].

### Nondenaturing gradient gel electrophoresis and western blotting

Microdialysis, CSF, or microdialysis buffer samples were diluted with Native PAGE sample buffer (Life Technologies, Carlsbad, CA, USA) and electrophoresed on a 4-20% Tris-Glycine gel (Life Technologies, Carlsbad, CA, USA) at 100V for 24 hours at 4°C. A mixture of proteins with defined hydrated diameters was used for size standards (Amersham™ HMW Calibration Kit for Native Electrophoresis, Cat. # 17-0445-01, GE Healthcare). Proteins were transferred to a nitrocellulose membrane and probed with antibodies recognizing goat anti-human apoE (50A-G1b, 1:200, Academy Biomedical). Membranes were then washed extensively and probed with a donkey anti-goat IgG conjugated to HRP (1:5000, sc-2056, Santa Cruz Biotechnology). Blots were then developed using enhanced chemiluminescence (Lumigen-TMA6, Lumigen Inc.) and imaged with a Syngene G-Box (Syngene, Cambridge, UK).

### Statistical analysis

Statistical analysis was performed using PRISM version 5.0 (GraphPad). Data are presented as mean ± SEM. Comparisons between two groups were made using a two-tailed unpaired t-test. Multiple groups were compared using ANOVA followed by Tukey’s *post hoc* test. Statistical significance was assigned to p-values less than 0.05.

## Competing interests

DMH co-founded and is on the scientific advisory board of C2N Diagnostics and currently serves as a consultant for Astra Zeneca, Bristol Myers Squibb, and Genentech. GEL co-founded ReXceptor, Inc.

## Authors’ contributions

JDU, GEL, JRC, JMC and DMH conceived and designed the experiments. JDU, JMC, TEM and DRS performed microdialysis experiments. JDU, JMC and JMB analyzed microdialysis samples. JLR, JMB, and JRC performed bexarotene experiments. HJ prepared CSF samples. JDU and PBV performed native gel electrophoresis analysis. JDU wrote the paper and JRC and DMH revised the paper. All authors gave final approval of the version to be published. All authors read and approved the final manuscript.
